# Study on the Differential Value of Tumor Marker CA724 on Primary Gastric Cancer

**DOI:** 10.1155/2021/2929233

**Published:** 2021-09-25

**Authors:** Jieying Ding, Han Zhang, Zixian Wu

**Affiliations:** Department of Clinical Laboratory, Shanghai Ninth People's Hospital, Shanghai Jiao Tong University School of Medicine, Shanghai 200011, China

## Abstract

We investigated the diagnostic value of the tumor marker CA724 in patients with primary gastric cancer. One hundred forty-six patients with primary gastric cancer were selected as the observation group; 89 patients with gastritis treated in the same period were included in the control group 1; 91 patients with healthy physical examination during the same period were included in the control group 2. Electrochemiluminescence immunoassay was used to determine the level of carbohydrate antigen CA724 in each group; the pathological data of the observation group were consulted, and the expression level of tumor marker CA724 under different pathological conditions was analyzed; ROC curve was drawn to evaluate the diagnostic value of CA724 in gastric cancer and gastritis. The level of CA724 in primary gastric cancer patients was significantly correlated with tumor diameter, tumor stage, differentiation type, and lymph node metastasis. The ROC curve was drawn with a CA724 cutoff value of 7.94 U/Ml. The AUC value of CA724 in primary gastric cancer patients was 0.815, with a diagnostic sensitivity of 84.68% and a specificity of 71.95%. In conclusion, CA724 was highly expressed in patients with primary gastric cancer, which can achieve the diagnostic differentiation of gastric cancer and gastritis, and obtain a high diagnostic efficiency, providing a reference basis for clinical diagnosis and treatment.

## 1. Introduction

Gastric cancer is a malignant tumor originating from the epithelium of the gastric mucosa, which is more common in people over 50 years of age and has a slightly higher incidence in men than in women. In recent years, the incidence of gastric cancer has been on the rise due to changes in people's diet, increased work pressure, and *Helicobacter pylori* infection [1]. Previous studies have reported that gastric cancer can occur in any part of the stomach, with most patients occurring in the sinus, greater curvature, and lesser curvature of the stomach [2]. Most patients with gastric cancer are adenocarcinoma, and the symptoms are not obvious in the early stage of development. With the prolongation of the disease, it can be accompanied by nonspecific symptoms such as upper abdominal discomfort and belching, and it is similar to gastritis, which makes clinical diagnosis and treatment more difficult [3]. Although pathological tissue examination is the “gold standard” for diagnosing primary gastric cancer, it can help patients to confirm the diagnosis, but it is difficult to be applied in primary hospitals because of the high diagnostic risk [4, 5].

Tumor marker CA724 is a glycoprotein antigen, with a double antigenic determinant cluster, mainly found in human adenocarcinoma tissues, and is considered a tumor marker in the gastrointestinal tract and ovaries [6]. Previous studies have shown that CA724 is highly expressed in solid tumors such as gastric, breast, and lung cancers, reflecting the severity of the disease [7]. However, the application of CA724 in primary gastric cancer and gastritis has been less investigated. Therefore, this study was conducted to investigate the diagnostic value of CA724 in patients with primary gastric cancer and gastritis.

## 2. Materials and Methods

### 2.1. Clinical Data

146 patients with primary gastric cancer who underwent surgery at Shanghai Ninth People's Hospital, Shanghai Jiao Tong University School of Medicine, Shanghai, China, from January 2018 to December 2020 were selected for the study and also set up as an observation group. The clinical data for the patients were as follows: 84 males and 62 females, aged 37–84 years old, mean: 61.49 ± 6.61 years; disease duration: 1–13 months, mean: 6.93 ± 0.85 months; tumor diameter: 1–6 cm, mean: 3.41±0.89 cm; clinical stage: 81 cases of stages I-II, 65 cases of stages III-IV; differentiation type: 33 cases of low differentiation, 71 cases of medium differentiation, 42 cases of high differentiation, and 51 cases of lymph node metastasis. 89 patients with gastritis treated at the same time were selected as control group 1, 49 men and 40 women, aged 36–85 years old, average, 60.98 ± 6.58; the duration of the disease was 1–12 months, with a mean of 6.91 ± 0.83 months. 91 patients with health check-ups at the same time were selected as control group 2, 53 males and 38 females, aged 35–84 years, with a mean of 60.15 ± 6.51 years. This study was approved by the Ethics Committee of the Shanghai Ninth People's Hospital, Shanghai Jiao Tong University School of Medicine, Shanghai, China. All patients provided written informed consent.

### 2.2. Inclusion and Exclusion Criteria

  Inclusion criteria: (1) patients in the observation group met the diagnostic criteria for primary gastric cancer [8] and were diagnosed by pathological tissue examination; (2) control group 1 met the diagnostic criteria for gastritis and was diagnosed by gastroscopy; (3) everyone had completed the CA724 test and could tolerate it; (4) complete baseline and follow-up data.
  Exclusion criteria: (1) patients with mental disorders, cognitive dysfunction, or other malignant tumors; (2) patients with autonomic nervous system diseases and severe liver and kidney dysfunction; (3) patients who had received radiotherapy and chemotherapy before the examination and had autoimmune system diseases.

### 2.3. Methods

#### 2.3.1. Specimen Collection

3 mL of peripheral fasting blood was obtained from the patients in the observation group and control group 1 the next day after admission and from the patients in the control group 2 on the day of healthy physical examination. The blood was centrifuged for 10 minutes at a speed of 3000 rpm and stored at a low temperature for further use.

#### 2.3.2. Detection Method

Electrochemiluminescence immunoassay (Roche Cobas e80, Roche, Switzerland) was used to determine the level of CA724 in each group. The pathological data of the observation group (including gender, age, tumor diameter, tumor stage, differentiation type, and lymph node metastasis) were checked, and the expression levels of CA724 under different pathological conditions were evaluated [9, 10].

#### 2.3.3. ROC Curves

ROC curves were drawn to analyze the diagnostic value (diagnostic sensitivity and specificity) of CA724 in gastric cancer and gastritis.

### 2.4. Statistical Analysis

The statistical analysis was performed by SPSS24.0 software and expressed by *n* (%). The *t*-test was used for comparison between groups, and the *χ*^2^ test was used to compare all counting data between groups. The difference was statistically significant when *p* < 0.05.

## 3. Results

### 3.1. Comparison of CA724 Levels in the Three Groups

The patients in the three groups all completed the investigation of CA724 levels. As given in Table 1, the results showed that there was no significant difference in the level of the CA724 in control group 1 and control group 2 (*p* > 0.05); in the meantime, the CA724 levels in the observation group were higher than those in the control group 1 and control group 2.

### 3.2. Comparison of CA724 Levels in the Observation Group under Different Pathological Conditions

The pathological data of all patients were collected, and the results showed that the level of CA724 in patients with primary gastric cancer was not statistically correlated with gender and age (*p* > 0.05). At the same time, it was statistically related to tumor diameter, tumor stage, differentiation type, and lymph node metastasis (*p* < 0.05, Table 2).

### 3.3. ROC Curve of CA724 in Patients with Primary Gastric Cancer

**Figure 1:**
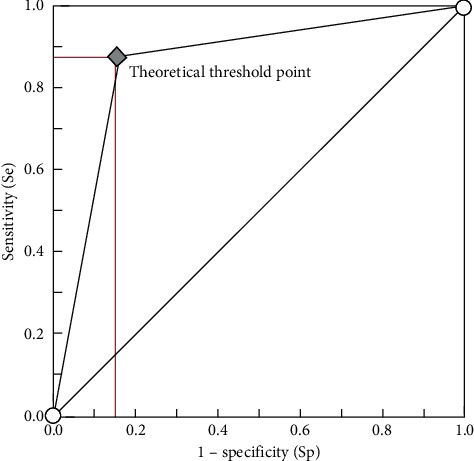
ROC curve of CA724 in patients with primary gastric cancer.

The ROC curve was drawn with the cutoff value of the CA724 at 7.94 U/mL. The results showed that the AUC value of the CA724 for patients with primary gastric cancer was 0.815, the diagnostic sensitivity was 84.68%, and the specificity was 71.95% (Figure 1).

## 4. Discussion

Primary gastric cancer is a malignant tumor with high clinical incidence, and with the change of people's lifestyle, it has led to an increasing trend of disease incidence [11]. However, the early diagnosis rate of primary gastric cancer is relatively low, leading to a high clinical mortality rate, and it is clinically important to choose an appropriate diagnostic method to improve the detection rate and patient prognosis [12]. Histopathological examination is a common diagnostic technique to differentiate primary gastric cancer and gastritis, and it is considered the “gold standard” for diagnosis. However, this diagnostic approach is risky, invasive, and requires high instrumentation and equipment, so it is difficult to apply it at the grassroots level [13].

With the improvement and development of medical technology, clinical research in the area of tumor markers has been strengthened. Tumor markers are chemical analogs that reflect the presence of tumors and are expressed at low or no levels in normal tissues and are present only in embryonic tissues. However, their levels are significantly higher in tumor tissues [14, 15]. Previous studies have shown that the existence and quantitative changes of tumor markers can reflect the nature of the tumor to some extent and can reflect the histogenesis, cellular function of the tumor, and thus the severity of the patient's disease [16]. A series of studies have explored the diagnostic and prognostic value of various serum tumor markers in gastric cancer [17, 18]. Tumor biomarker CA724 trended to be considered as an independent prognostic factor [19]. Tong et al. found that CA724 predicted overall survival in locally advanced gastric cancer patients with neoadjuvant chemotherapy [20]. In this study, there was no significant difference between the levels of tumor marker CA724 in control group 1 and control group 2 (*p* > 0.05), while the level of tumor marker CA724 in the observation group was higher than that in both control group 1 and control group 2 (*p* < 0.05). Taken together, we found that tumor marker CA724 was highly expressed in patients with primary gastric cancer. At the same time, it had lower levels in patients with gastritis, suggesting that strengthening CA724 level measurement can achieve the diagnosis and differentiation of primary gastric cancer and gastritis.

CA724 is a gastric cancer antigen, one of the laboratory indicators for detecting gastric cancer and various gastrointestinal cancers, and it is a nonspecific tumor marker. Previous studies have shown that elevated levels of CA724 do not indicate that patients have tumors [21]. They are more common in gastrointestinal tissues and have high sensitivity to gastric cancer and nonsmall cell lung cancer. In this study, the level of the tumor marker CA724 in patients with primary gastric cancer was significantly correlated with tumor diameter, tumor stage, type of differentiation, and lymph node metastasis (*p* < 0.05), indicating that the elevated expression level of CA724 in patients with primary gastric cancer could reflect the severity of the disease. Previously performed studies have reported that CA724 is a second-generation tumor-associated glycoprotein-72 with dual antigenic determinants, and its expression level is elevated in malignant tumors such as gastric cancer, colon cancer, and lung cancer [22]. However, the increase in the level of CA724 does not mean that the patient must have a tumor disease. The level of this indicator is also elevated in type 2 diabetes, liver cirrhosis, rheumatism, and gastrointestinal disorders. Studies have shown that CA724 is a sugar chain antigen that can act as a cell surface adhesion molecule to participate in the occurrence and development of tumors [23]. In order to further analyze the diagnostic value of CA724 in primary gastric cancer and gastritis, the ROC curve was drawn in this study with a CA724 cutoff value of 7.94 U/mL. The results showed that the AUC value of CA724 in primary gastric cancer patients was 0.815, with a diagnostic sensitivity of 84.68% and a specificity of 71.95%, indicating that CA724 can achieve the diagnostic differentiation between primary gastric cancer and gastritis, and has high diagnostic efficacy. Therefore, clinical attention should be paid to CA724 levels when they are elevated, and other methods of diagnosis should be combined when necessary to help patients with early diagnosis [24].

## 5. Conclusion

CA724 was highly expressed in patients with primary gastric cancer, which can achieve the diagnostic differentiation of gastric cancer and gastritis, and can obtain a high diagnostic efficacy and provide a reference basis for clinical diagnosis and treatment. In future clinical practice, tumor marker CA724 can be applied to diagnose primary gastric cancer to improve the diagnostic accuracy and provide timely and effective treatment for patients.

## Figures and Tables

**Table 1:** Comparison of CA724 levels in the three groups.

Group	Cases	CA724 (U/mL)
Observation group	146	52.53 ± 5.39^#*∗*^
Control group 1	89	4.51 ± 0.64
Control group 2	91	4.42 ± 0.61
*F*	—	7.195
*P*	—	

^#^Compared with the control group 2, *p* < 0.05; ^*∗*^compared with the control group 1, *p* < 0.05.

**Table 2:** Comparison of CA724 levels in the observation group under different pathological conditions ($\bar{x}\pm s$).

Pathological type		Cases	CA724 (U/mL)	*t*	*P*
Gender	Male	84	51.98 ± 5.35	1.593	0.425
	Female	62	53.87 ± 5.51		

Age (years)	≥60	79	51.90 ± 5.31	0.615	0.893
	<60	67	53.89 ± 5.54		

Tumor diameter	≥3 cm	73	79.69 ± 8.51	9.154	≤0.001
	<3 cm	73	43.19 ± 5.12		

Tumor stage	I-II	81	46.96 ± 5.31	8.391	≤0.001
	III-IV	65	80.44 ± 8.78		

Differentiation type	Poorly differentiated	33	91.45 ± 9.51	11.691	≤0.001
	Moderate differentiation	71	54.69 ± 6.13		
	Well differentiated	42	40.31 ± 4.96		

Lymph node metastasis	Yes	51	94.59 ± 9.15	10.529	≤0.001
	No	95	42.33 ± 4.29		

## Data Availability

The datasets used and/or analyzed during the current study are available from the corresponding author upon request.
